# Integrating drones into NHS patient diagnostic logistics systems: Flight or fantasy?

**DOI:** 10.1371/journal.pone.0264669

**Published:** 2022-12-22

**Authors:** Andy Oakey, Matt Grote, Angela Smith, Tom Cherrett, Aliaksei Pilko, Janet Dickinson, Laila AitBihiOuali

**Affiliations:** 1 Faculty of Engineering and Physical Sciences, University of Southampton, Southampton, United Kingdom; 2 Bournemouth University Business School, Bournemouth University, Bournemouth, United Kingdom; National Taiwan University of Science and Technology, TAIWAN

## Abstract

Healthcare accounts for approximately 5% of emissions in developed nations, and the public healthcare provider in the United Kingdom (UK), the National Health Service (NHS), has set a target to reach net-zero emissions by 2040 without detriment to its quality of patient care. With Uncrewed Aerial Vehicles (UAVs; a.k.a. drones, UAS, or RPAS) starting to be used in healthcare systems outside the UK, there is interest in how they could be integrated into NHS operations to transport diagnostic specimens. Reflecting on a business-as-usual analysis of current NHS diagnostic specimen logistics across the Solent region (southern UK), this paper critically evaluates the practical reality of integrating UAV deliveries of this commodity, identifying the benefits and challenges that must be addressed to realise commercial services, including dangerous goods legislation, cargo stability, routing, and weather. In the analysis, 14 out of 79 surgeries could be realistically served by a 5m wingspan vertical take-off/landing (VTOL) UAV: seven directly, and seven via ground-based transfers. The results suggested that an average of 1,628 samples could be served by UAV each week, resulting in 42 flights/week with 10 taxi services to cover periods where weather limited flying. This equated to an approximate total service cost of £2,964/week if regulations develop to relax UAV personnel constraints. The introduction of UAVs reduced the marginal external costs (greenhouse gas emissions, congestion, and air pollution) by £196 per week and cut travel times to UAV served sites by 72% (weather permitting). Tailpipe emissions (excl. taxis), vehicle-kilometres travelled, and van costs were reduced by 20%, 20%, and 23% (respectively), but the overall system cost increased by 56%. Whilst this increase is likely to make the introduction of UAV services financially challenging, the benefits in terms of emissions and journey time savings may offset some of the additional cost and warrant further investigation.

## Introduction

Valid use cases for Uncrewed Aerial Vehicles (UAVs; a.k.a. drones, unmanned aircraft systems–UAS, or remotely piloted aircraft systems–RPAS) within healthcare logistics are being increasingly explored [1], with many initial trials undertaken in developing countries [2]. The scope for UAVs to overcome the challenges of transporting diagnostic specimens to laboratories from remote rural communities in South Africa as part of tuberculosis and malaria treatment programs was first recognized in 2007 with trials of the e-Juba UAV (electronic pigeon), [3]. Commercial UAV operations have since emerged, with Zipline operating a network of UAVs to distribute vaccines, medicines, and blood products to more than a thousand medical facilities across the state of Kaduna in Nigeria and also operating a nationwide service for the distribution of blood products in Rwanda [4,5]. UAV healthcare logistics operations are now emerging in developed countries, and there is growing interest in the United Kingdom National Health Service (NHS) as to what benefits might be gained from the introduction of UAVs in terms of (i) improved patient care; and (ii) emissions reductions to help the service become net-zero by 2040 [6]. Using a significant data set of historic patient diagnostic sample collections from the 79 surgeries across Southampton UK, this paper investigates the potential benefits, financial and practical realities of integrating UAVs into existing van-based logistics fleets.

## Literature review

Where road infrastructure is poor and topology challenging, UAVs have been shown to offer significant journey time advantages compared to traditional road transport modes [7]. Often, airspace is less congested in developing countries and with more sparsely populated areas, UAV operations present fewer safety concerns to regulators which has led to such regions becoming test beds for UAV services [8]. The integration of UAVs into medical logistics in more developed countries has been somewhat slower, largely resulting from the strict airspace management legislation which dictates that beyond-visual-line-of-sight (BVLOS) flying requires specific permissions which are often granted to the detriment of other airspace users via temporary restricted flight corridors (Temporary Danger Areas, TDAs in the UK) [9]. In November 2020, Matternet announced their intention to implement routine UAV services to support pathology services across a network of hospitals in Berlin [10], but despite aspirations for wider development, very few commercial services exist. Other applications in medical logistics include DHL’s parcelcopter, which performed deliveries of medication to remote islands in Germany, 12km from the mainland, using a vertical take-off and landing (VTOL) platform in 2014 [11], while Skyports, using Swoop Aero’s VTOL platform, have delivered patient diagnostics from remote Scottish island communities to mainland pathology labs in the UK in 2021 [12,13]. Despite these successful trials, few services have moved beyond extended demonstrators to the point of commercialisation, suggesting that wider barriers to full implementation of UAV deliveries exist [14].

UAVs operate in many different configurations (electric or fossil-fuelled powered fixed-wing, multi-copter (capable of VTOL), or a hybrid of these arrangements (VTOL with fixed-wing cruise), with a range of options for collecting and delivering cargo such as cable systems, parachute drop, or integral cargo holds within the main fuselage [12,15,16]. With respect to performance, fixed-wing platforms are generally more efficient over longer distances compared to VTOL multi-copter UAVs but typically require runways to take-off and land [17]. From an NHS perspective, point-to-point UAV services not requiring runways would be essential due to space limitations around medical facilities; thus, VTOL platforms (multi-copter or hybrid) are favoured because of their flexibility.

UAV applications in medical logistics have been primarily based around low weight/volume, time critical items such as patient diagnostics (samples most typically of either blood, urine or stools), pharmacy related products (including chemotherapy), and bloods for transfusion [1]. In the case of the latter, Zipline’s country-wide blood service in Rwanda is making in excess of 150 flights per day [15], with each catapult-launched, fixed-wing UAV capable of carrying a 1.75kg load up to 120km at 100km/hr BVLOS between distribution centres and hospitals/clinics, where they deliver via parachute [18–20]. The speed of service is the key benefit, with Zipline reporting a 95% reduction in blood waste and spoilage [21]. This is largely due to the difficult nature of the terrain and the condition of the road infrastructure which can make journey times by land logistics unreliable. It should be noted that the cost-effectiveness of this system has not yet publicly demonstrated or explored, with the medical improvement being the primary aim so far in its development [22]. A cost estimation based on the company’s operations in Ghana has suggested each flight costs approximately $17 on average; notably more expensive than other modes [4]. It is understood that the service is heavily subsidised by the government so that healthcare providers can afford to use it, meaning that long-term use would become unsustainable if funding was removed [4].

Of increasing interest is whether diagnostic specimens (commonly referred to as ‘pathology’ or ‘laboratory samples’ or ‘specimens’) could be more efficiently moved for the NHS by UAV, particularly between more remote surgeries and analysis laboratories. Specimens are routinely taken from patients by clinicians with an estimated 80% of treatment plans following on from some form of diagnostic sample analysis [23]. After being taken, samples must be stored and transported to the analysis laboratory in a controlled manner, where they must be analysed within a specific time frame [24]. The NHS is in the process of implementing a national ‘hub-and-spoke’ style diagnostic specimen collection system across a series of 29 networks [25,26]. Inter-hospital and GP surgery-to-hospital transport is typically handled by multiple vehicle rounds [27] which are subcontracted to third-party logistics providers who provide dedicated collection services for patient diagnostic samples from each surgery on a daily basis along with the replenishment of fresh specimen tubes, associated ancillary packaging and paperwork.

Local area diagnostic specimen collections in the UK are typically undertaken using dedicated logistics providers, operating routine collection services using multiple vehicles linking General Practitioner (GP) surgeries (also known as clinics or doctor’s offices in other countries) to pathology laboratories where the samples are analysed [28]. As an example, for the catchment area around Southampton, a city on the South coast of the UK with population ~250,000, there are 79 GP surgeries generating some ~3,000 samples each weekday (~70,000 samples/month), which are collected using 10 dedicated van rounds, each servicing between 4 to 27 surgeries per day, with many surgeries visited twice daily. Surgeries typically batch samples together for a morning and afternoon collection using standardised packaging approved by the Medicines and Healthcare Products Regulatory Agency (MHRA). A key drawback with this system is that, in most cases, vans do not visit the pathology lab until 11:00 AM at the earliest, which results in significant workload for the analysis teams at specific times of the day [29]. One school of thought is that UAVs could be used to target particularly remote surgeries which contribute significantly to the overall carbon footprint of the van round whilst also bringing in batches of samples for earlier analysis. This may also lead to faster bleed-to-diagnosis time for patients.

During the first year of the COVID-19 pandemic, it was identified that samples collected from GPs were turned round significantly slower compared to hospital generated samples with less than a third of community tests being turned around the same day, as opposed to 80% in hospital [30]. The implementation of UAVs into existing logistics systems may offer the potential to alleviate some of these issues with trials in the Argyll and Bute region of Scotland by Skyports reportedly saving over 12,000 hours of waiting time in a 90 day operational period involving 14,000 km of BVLOS flights compared to the business-as-usual system [31]. This case study was ideally suited to UAV deployment where island communities could be more effectively served using a point-to-point air bridge as opposed to dual mode van and ferry-based services that are potentially less flexible.

Comtet et al. [32] investigated the potential for UAV delivery of laboratory samples, primarily from the management and regulatory perspective, and noted that there were concerns with respect to the flexibility in arrangement of routes when integrating UAVs. For example, changes on a van route would be easily achieved by adding/reordering stops, whilst a UAV service would likely require additional trips. Challenges in terms of the loads carried by current rounds were also presented, with weight and size variations being cited as a problem area but the potential for improved travel times and distances was cited as a significant benefit of the proposed system.

Koshta et al. [33] also investigated the challenges of implementing UAVs in rural healthcare supply chains and found that the most significant barrier to implementation related to a lack of government regulations, impacting the ability to undertake BVLOS flights. They also found that carrying capacity, range, and weather proofing were also potential barriers in terms of the readiness of the UAV technology itself.

Although UAVs have demonstrated benefits to medical logistics operations in Africa, they have not progressed beyond limited short-term trials in developed countries, and some of the major logistics players have scaled-down their UAV service development, suggesting there may be wider challenges which will limit their commercialisation [14,34–36]. Some of the key issues that have to be overcome before widespread BVLOS UAV operations are realised include: i) agreeing and implementing a system of air traffic control such that UAVs and crewed aircraft can operate in shared air space; ii) understanding the implications on UAV design resulting from the specific carriage regulations related to different medical products (including dangerous goods carriage regulations); iii) determining routings for UAVs that minimise air and ground risks but do not negatively impact on range, particularly for battery powered UAVs; iv) satisfying the medical product regulatory agencies (e.g. the MHRA) that the stability of medical products will not be adversely affected through exposure to excessive vibration or temperature during carriage by UAVs; v) developing reliable contingency strategies for periods when adverse weather prevents UAV flights [34,37,38].

All of these elements add uncertainty when attempting to calculate the true costs of UAV operations in both urban and rural settings, and there have been some attempts to do this for retail UAV logistics. Jenkins et al., (2017) attempted to cost business-to-consumer (B2C) package deliveries via a VTOL UAV where the package weighed under 5 pounds [39]. Costs were taken from 25 commercial UAV operators and were determined to be:

- *Battery costs*—$100 (to power a UAV carrying 5 pounds (2.26 kg), 10 miles (16km))
- *Battery life*– 250 hours
- *Motor costs*—$60 (for each motor, with 4 being required to fly a 10-pound UAV 6 miles)
- *Motor life*– 750 hours
- *Rotors*—$1 (wholesale price for a set of four commercial grade rotors)
- *Marginal electricity cost*—$0.25 per flight

In terms of hourly operational costs, these were estimated to be $0.94 made up of: Insurance ($0.02/hr); Command and control ($0.02/hr); Communication ($0.02); Labour ($0.02/hr); Maintenance ($0.40/hr batteries, $0.08/hr motors, $0.01/hr rotors, $0.03/hr electrical); Battery charging ($0.24/hr); Airspace charges ($0.10/hr). Jenkins et al., estimated that if an individual UAV platform cost of $2000 was assumed, with each UAV being able to fly a minimum of 50 hourly flights per week, the cost per trip would be $1.74 against $2.50 per typical last-mile delivery using traditional van-based delivery methods [39]. This was under the overall assumption that between 86 and 91% of the 86 million packages generated in the U.S. daily were less than 5 pound in weight and could be serviced via UAV. The authors also assumed that operators would replace all components at least once per year due to wear-and-tear.

A key unknown is how much access to airspace will cost UAV operators, and how this will be managed on a large scale. The concept of UAV Traffic Management (UTM) has been developed as a solution to integrating UAVs and crewed aircraft in low-altitude airspace (under 400 ft) and would be operated by any number of UAS Service Suppliers (USS) [40]. The system would require UAV operators to adopt various equipment, conforming to specific standards in relation to UAV tracking and remote identification, UAV-to-UAV communication and detect-and-avoid sensor technology. These would all have a cost associated with them with elements being potentially provided by the USS for a monthly/annual subscription fee. Bohlig (2017) estimated that in the U.S., this could be in the region of $200-$300 per UAV per annum [41].

Weather conditions may also be a limiting factor in the use of UAVs. In Winter 2019/20, the UK experienced seven named storms; a system used to highlight weather of significant severity [42,43]. London’s Heathrow Airport experiences strong winds on 60 days in a typical year, with ‘strong winds’ defined as averaging gusts ≥ 28 knots (14.4 m/s); cumulonimbus (CB) cloud warnings, often associated with disruptive weather, on 10 days per year; and snow/ice warnings on 5 days per year [44–46]. In such weather, it is likely that UAVs will either not physically be able to achieve their planned flights, due to concerns around flight stability, energy limitations, or regulators limiting their use for reasons of safety.

A study by Gao et al. (2021) defined a weather resistant UAV as being able to fly in 14 m/s winds and precipitation of up to 50 mm/h. It was highlighted that at latitudes covered by the UK, winds were typically flyable for the majority of the year, with the greatest proportion of weather impacts featuring in spring months (Mar-May); however, the positioning of the UK relative to the Atlantic Ocean meant that it is exposed to the North Atlantic Oscillation and stronger winds than other areas of similar latitude as a result [38,47]. Jenkins et al., (2017) examined the potential impact of weather on UAV operations using three years’ worth of historic weather patterns around Salt Lake City International Airport. The results suggested that over the three-year period, there were only 27 occurrences where the mean wind speed exceeded 30 mph for a 10-minute interval and 138 days where wind speeds were between 15 and 30 mph.

Much interest has been shown in how UAVs might make NHS logistics operations more efficient and timely but the potential costs versus the benefits have not been fully investigated beyond short-term trials. Given the volumes of patient diagnostic samples to be collected on a daily basis, it is unrealistic to expect that such an undertaking could be completed by UAVs alone, particularly given the difficulties of gaining permissions to fly over populated areas where vans or cargo cycles could prove more efficient. More realistic is to think of UAVs serving remote or outlying surgeries that take up considerable van-time to service in the business-as-usual scenario. In this way, UAVs might complement existing van fleets to provide a speedier service to remote outposts.

This paper investigates these issues and attempts to quantify the potential costs and benefits of integrating UAVs into an existing patient diagnostic service serving the area around Southampton and the New Forest compared to the business-as-usual van-based collection rounds.

## Methodology

As part of two funded research projects (E-Drone, [48]; Future Transport Zone, [49]), an audit of existing logistics activity related to patient diagnostic collections was undertaken, working with the NHS Trusts in the Solent Region of the UK, and specifically, Southampton General Hospital (SGH) located within the city of Southampton. A historical dataset was obtained of patient diagnostics movements from 79 GP surgeries (also known as ‘doctor’s offices’) in and around Southampton (for November 2018 and March 2021 constituting ~70,000 sample movements/month) to the pathology analysis laboratories at SGH. This dataset was analysed to identify: i) the mean, minimum and maximum flows of products between the various origin and destination points by collection vehicle type; ii) the GP surgery origin locations that impact most on BAU van round time and could be suitable for UAV integration; and iii) the implications on UAV carriage requirements resulting from the range, weight and number of products needing to be transported. The typical round schedules from September 2018 were also provided, enabling a comparison to the business-as-usual case.

The audit also involved a series of informal discussions with practice and logistics managers overseeing the day-to-day operations of the patient diagnostic services at SGH to accurately understand the current transportation procedures, and opportunities. The practical experiences gained from undertaking the first BVLOS trial of a fixed-wing UAV across the Solent (09/05/20) during the first Covid-19 lockdown [50] and subsequently from mainland UK to the Isles of Scilly (15/12/20) involving the authors [51,52] were also used to reflect on the realities of transitioning UAV services to integrate with land logistics operations in terms of flight permissions, goods carriage legislation and maintaining cargo integrity.

The business-as-usual data collected through the audit were then used in a desktop analysis to quantify the likely numbers, timings and costs of flights that would be needed to provide a UAV collection service for patient diagnostic samples generated by GP surgeries deemed suitable for service by UAV out of the existing 79. The analysis also set out to evaluate the potential benefits to the NHS in terms of improved service times and reduced transport related CO_2_.

To understand the flight timetabling requirements, the sample production rates from the historic data were used with a maximum capacity per flight, identifying the time points in each day when either (i) a full load was ready; or (ii) the last sample of the day had been taken at each site served by UAV.

### Units of carriage used in the desktop analysis

**Fig 1:**
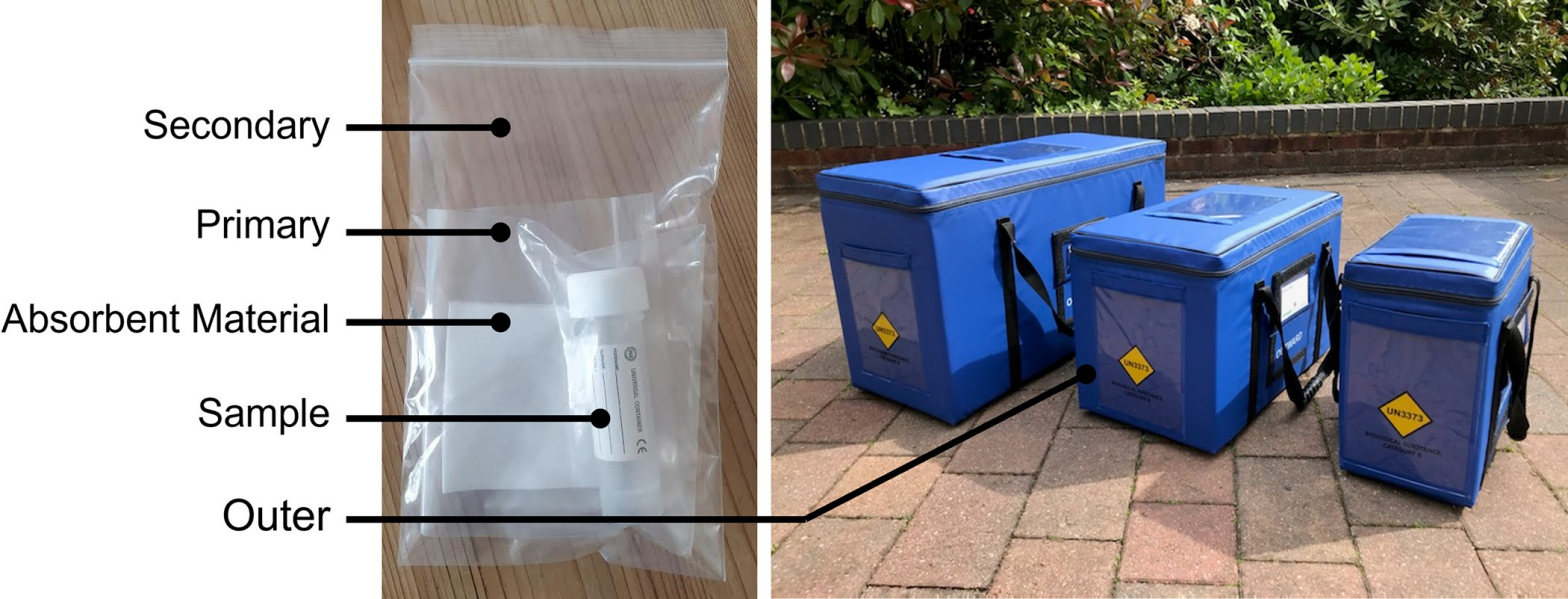
UN3373 PI650 compliant packaging. Left–sample, contained in leakproof primary and secondary packaging with absorbent material; right–Versapak insulated outer packaging (centre (‘medium’) = 460mm (w), 255mm (D), 305mm (H)).

In the NHS, patient diagnostic samples are typically taken at local GP surgeries and are transported at ambient temperature in specimen containers (Fig 1) within three-layer packaging according to the PI650 packaging instruction under UN3373 (Biological Substance Category B—Diagnostic Specimens) carriage regulations to analysis laboratories which generally reside in major hospitals [25,53]. Some smaller hospitals also have facilities for basic pathology analysis and there are also cases where laboratories are operated by private companies such as Viapath in London’s Guy’s and St Thomas’ NHS Foundation Trust [23,54]. In this paper, the medium Versapak (Fig 1, central container) routinely used by GP surgeries across Southampton was taken as the unit of carriage, with an assumed maximum load of 50 samples per Versapak.

In terms of dangerous goods classification, work by Grote et al., (2021) has highlighted that biological materials considered sufficiently low risk as infectious substances under hazard class 6 (UN3373) can avoid classification as dangerous goods for air transport when packaged in PI650 standard packaging (Fig 1) [55,56], and therefore can be managed in a similar way to when they are transported by ground transportation modes, conforming to the MHRA Good Distribution Practice (GDP) guidelines [57]. Civil Aviation Authorities are still developing regulations in this area, and applications for approval are largely handled case-by-case [58]. Recent trials and research have highlighted that medical logistics operators must demonstrate that transport by UAV would not adversely affect the quality and stability of the medical cargo as a result of in-flight conditions experienced during transit [52,56]. Current understanding of UAV flown diagnostics suggests that no significant damage is caused [59], though this may vary by platform, packaging, and product.

### UAV used in the desktop analysis

**Fig 2:**
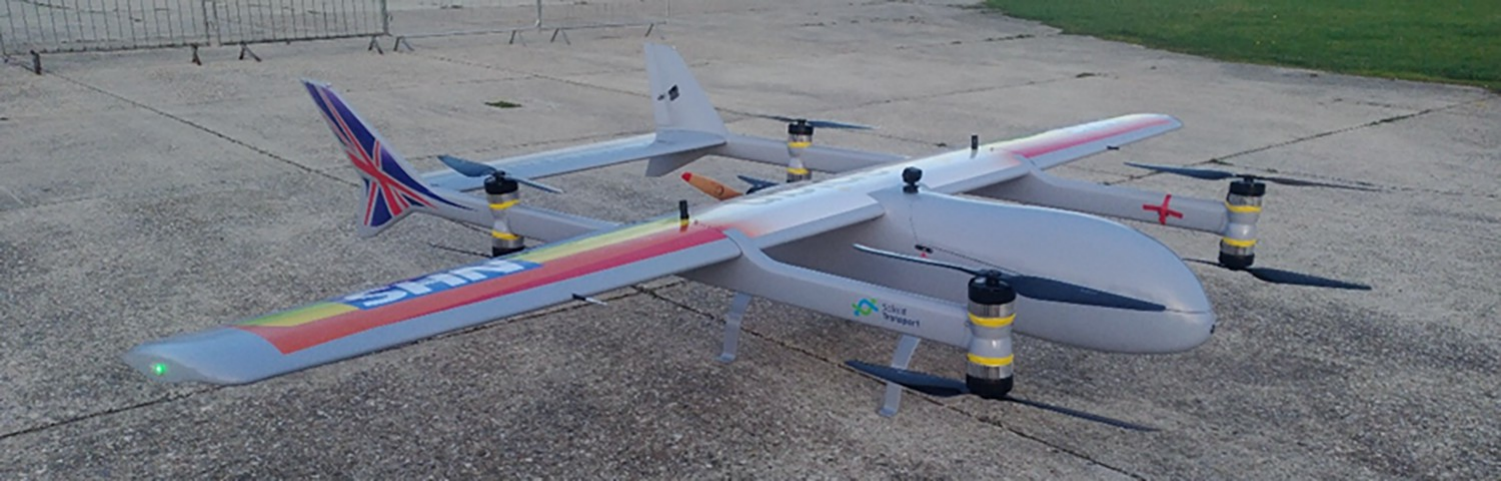
A render of a Mugin-5 Pro, 5m wing-span VTOL fixed-wing UAV, with a human for scale.

**Fig 3:**
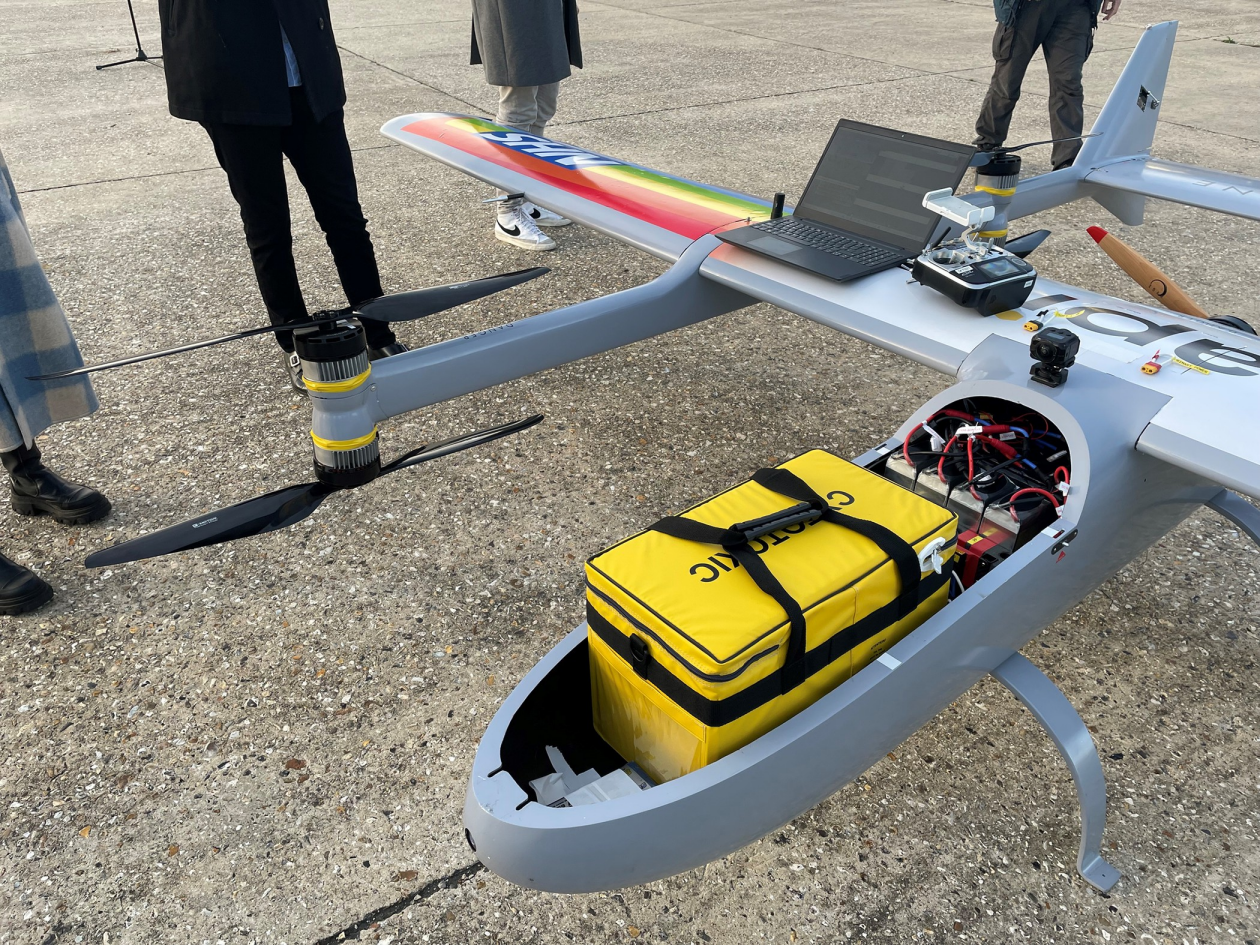
A medium sized Versapak (460mm (w), 255mm (D), 305mm (H)) loaded into the hold.

The reality of patient diagnostics logistics is that although the individual samples are small in terms of size and weight and ideal for transportation via UAV, they are consolidated into batches such that individual surgeries could be dispatching multiple Versapaks daily, each containing up to approximately 50 samples. In this situation, UAVs would have to have a payload capacity to accommodate at least one medium size Versapak ((460mm (w), 255mm (D), 305mm (H)), estimated capacity of ~50 samples) with an empty weight of approximately 2.4 kg (Figs 2 and 3) [60]. UAV trials involving such UN3373 cargoes (e.g. Skyports [31]) have been typically undertaken with small numbers of samples contained in bespoke packaging to fit the hold of the UAV. For any long-term commercial service, it would be necessary to carry the existing batch configurations as out-and-back flights using the standard Medicines and Healthcare Products Regulatory Agency (MHRA) approved packaging. An analysis of the sample production data suggested that of the approximate 3000 samples produced for collection each weekday across the 79 surgeries, the scale of production ranged from 1 sample at several surgeries, to 289 samples at the Coastal Medical Partnership (New Milton) surgeries which would have required 4–6 Versapaks, depending on actual sample size (mean per surgery = 41 samples/day, S.D. = 49 samples/day). Dialogue with the pathology lab practice managers highlighted that batches of samples from different surgeries cannot currently be mixed into consolidated loads unless undertaken in a controlled environment (e.g., a designated ‘primary surgery’).

UAV payload capacity has implications for both the planning of operations with aviation regulators in terms of the number of flights necessary and the routing, as well as the type of UAV platform needed. The practicalities of operating UAVs in terms of integration with land logistics systems must also be carefully considered, particularly where surgeries are located in built-up areas. Given the requirement in this research to carry a minimum of one medium sized Versapak from each surgery over the distances likely to be involved (~30 km+ one-way), a platform capable of carrying significant volume and weight (approx. 5kg min.) was required. To reduce land-take and infrastructure requirements for take-off and landing, the UAV would also need VTOL functionality, either as a fixed-wing hybrid platform or solely VTOL. Based on the authors’ experience of trials using a UAV carrying a Versapak in the Solent Region [61], and considering typical payload-wingspan relationships [62,63], a fixed-wing VTOL platform with a 5m wingspan (Figs 2 and 3) was deemed necessary to achieve carriage of such a load over practical distances (e.g., ~30km+) in variable wind conditions. The platform used in this paper was largely modelled on the characteristics of a Mugin-5 Pro 5m-wingspan VTOL platform.

### GP surgeries used in the desktop analysis

**Fig 4:**
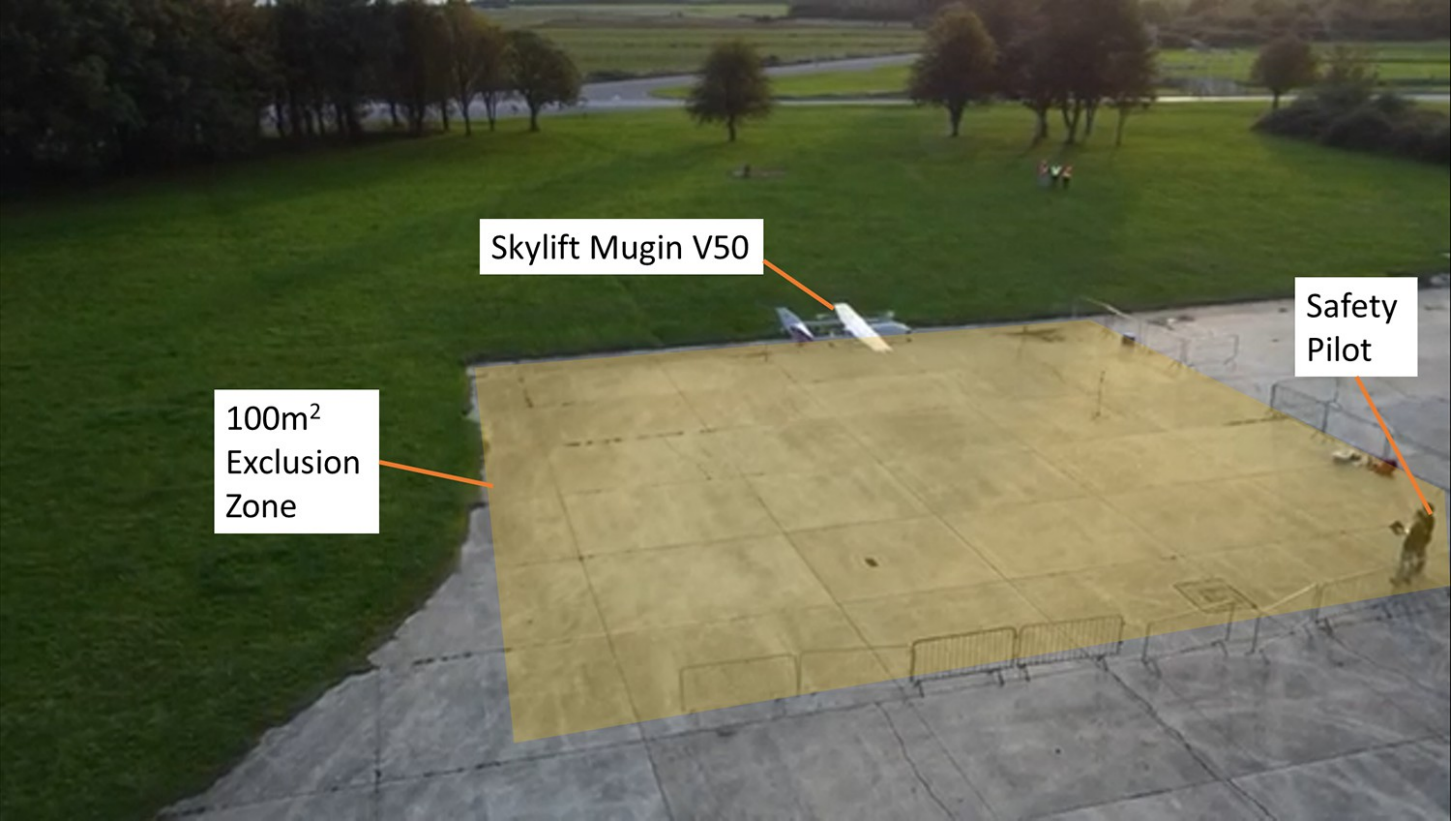
Example exclusion zone for take-off/landing, as used in flight trials for similar loads–approximately 100m^2^ [68].

Surgeries were deemed suitable if they: i) had a suitable UAV landing site in reasonable proximity (e.g., open space of approx. 100 m^2^ within the site grounds or on public land just outside (Fig 4) (note this differs from standard hobby UAV practice, but it was assumed rules could be slightly relaxed for trained operators [64,65]); ii) had a low ground risk UAV flightpath between the landing site and SGH (mean risk of a third-party fatality on the ground due to a UAV crashing along its flightpath = 1×10^−7^ fatalities/flight-hour or lower (100 fatalities per billion flight-hours)). For comparison, crewed aviation has a fatality risk of ~2×10^−5^ fatalities/flight-hour [66]); iii) were not in the flight path of Southampton Airport. Flight paths were planned using SEEDPOD [67], an open-source UAV risk route planning tool developed by the authors to minimise ground risk, with assessment based on the risk of fatalities due to a UAV crash on the ground under the flightpath. Airspace constraints in the immediate vicinity of Southampton airport were also considered in identifying suitable surgeries.

We also considered that secondary ‘satellite’ GP surgeries, in the vicinity of the primary surgeries, could be serviced by UAV, transferring their Versapaks of samples to the primary surgery by ground transport for consolidation and onward flight to SGH. Samples from satellite surgeries were assumed to be transferred by cycle courier to the primary surgeries, with catchment areas defined by the round-trip distance that could be cycled by a typical gig economy worker (e.g. Deliveroo operative) in approximately 20 minutes, plus a further 5 minutes accounting for service time [69].

The desktop analysis utilised the timings and volumes associated with every patient diagnostic collection during March 2021 across all of the 79 surgeries. The dataset recorded the date and time of day for each sample taken from a patient throughout the month. The UAV collection service was assumed to operate Monday-Friday, meaning the occasional samples taken on weekends (27 out of the 9,298 total samples generated at UAV-serviceable surgeries during the month) continued to be collected as per the existing BAU arrangements (i.e., collection by van). A medium size Versapak (empty mass 2.4 kg) with an assumed maximum capacity of 50 samples was used for collections, whilst the maximum payload of the UAV was assumed to be one full Versapak (up to 20 kg). UAVs were assumed to carry an empty Versapak on outbound journeys from SGH to the surgeries.

UAVs were assumed to make return trips servicing only one primary surgery at a time due to maximum payload constraints (i.e., one medium Versapak containing 50 samples), which ignores the possibility of larger UAVs with greater payload capacity visiting multiple surgeries per trip. Deliveries of samples to primary surgeries from satellite surgeries were assumed to be instantaneous (i.e., samples were all effectively taken at the primary surgery), ignoring the real-world practicalities of satellite-primary delivery schedules. Flight times were calculated based on a 21 m/s cruise speed.

### Operating strategy during inclement weather

Weather factors were considered in the analysis, with historic wind and precipitation data being used (provided by the MeteoStat Point API [70]). When a flight was calculated to be required, the weather for a 2-hour period following this was checked at the surgery in question, and at SGH. Should either site have wind speeds or precipitation levels outside of the assumed UAV operating range (10 m/s / 19.4knots and 50mm/h, referred to as a ‘common UAV’ with some rain resistance in [38]), the flight was cancelled and a taxi was used as a replacement for that surgery collection. Mean wind speeds at each site, and peak gusts at the hospital were used in the assessment of whether a UAV flight was possible. Data availability meant that only the hospital was used for gust analysis. Whilst winds will affect the ground speeds and durations of individual flights, it was assumed that return flights on the same path within a short time window would largely average out variations in travel speeds and times, simplifying calculations.

Due to surgeries continually producing samples even when weather conditions preclude UAV flights, the analysis accounted for contingency plans. Taxis were assumed as the transport mode to be used when weather conditions were deemed such that UAVs could not operate. This assumption was based on information from the discussions with NHS staff, where it was suggested that taxis were often used when other logistics options were not available to complete collections.

### Costings used in the desktop analysis

The cost estimates for the UAV collection service were derived from a combination of UAV development and design expertise within the research team and associated partners (www.cascadeuav.com), literature sources, and commercially available data [71]. Key cost elements that were sought related to: i) the life expectancy of specific UAV components (e.g., motors, propellers, servos, autopilots, communications equipment and the airframe itself); ii) the electrical energy consumed during the various stages of flight (take-off, transition, cruise, transition, landing); iii) human resource time for the personnel involved (mission commander, safety pilots, loaders); and iv) operational insurance (full breakdown in S1 Table).

There are likely to be additional fees for dangerous goods training and airspace management although these have not been included. Additionally, prices may be further inflated if a third-party operator is used, as a profit margin will need to be included in cost considerations.

The cost estimates used for collection by van, both in the BAU scenario and for non-UAV serviceable surgeries in the intervention scenario, were derived from the ‘Manager’s Guide to Distribution Costs’ (MGDC) published in the UK by the Freight Transport Association [72].

Delivery driver costs (£10.78/hour in S1 Table) were assumed to be the stated average value for drivers of light rigid vehicles (≤7.5 tonnes Gross Vehicle Weight; GVW), including overtime and productivity (i.e., bonuses for achieving productivity targets) pay. Vehicle operating costs (£0.46/mile in S1 Table) were assumed to be the stated value for a diesel van (≤3.5 tonnes GVW), based on average annual mileage (35,000 miles/year), and including insurance, vehicle tax, depreciation, fuel, tyres, maintenance, and overheads (e.g., salary of the transport manager, running the despatch office, etc.). For consistency, the CO_2_ emission factor used in the analysis (0.45 kg/mile for a diesel van ≤3.5 tonnes GVW) was also taken from the same source. Taxi fares were estimated using a local taxi firm quote tool [73].

Marginal External Costs (MECs, often known as society costs) were used to investigate the wider benefits of UAVs, including greenhouse gas emissions, congestion, and air pollution costs, based on government transport appraisal databook values [74–76]. The emissions of the UAV’s electricity consumption were assumed to be negligible.

## Results and discussion

The case study used was a simplified version of reality, in that the GP surgeries targeted were considered in isolation, ignoring the potential benefits of optimisation with other vehicles and synergies that might be available within the full NHS logistics system. Analyses of the results was based around a comparison against the BAU vehicle-based specimen collections. It should be noted that in this BAU dataset, vehicles also carried out ancillary activities (e.g., transporting internal mail, medical records, etc.) which are being phased out. As a result, an adapted BAU vehicle round structure was used in the comparisons.

### Business-as-usual patient diagnostic collection logistics

**Fig 5:**
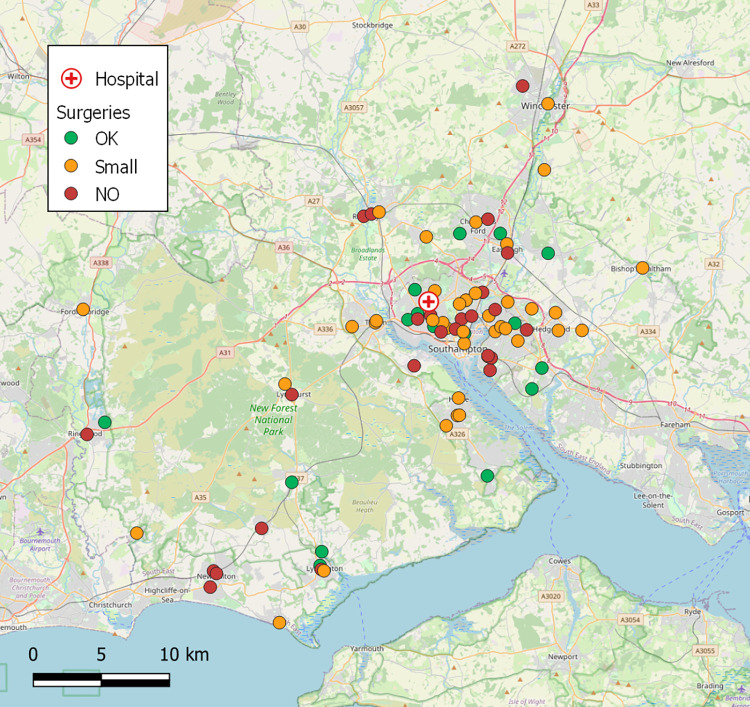
Locations of GP Surgeries in the Southampton area, coloured by landing site suitability. OK = suitable for a 5m fixed-wingspan craft to land with a reasonable safety buffer and existing facilities not significantly impacted (e.g., qty. of car park spaces taken). Small = suitable for 2m VTOL UAV. No = not suitable for a UAV of any size. Only landing site considered, risk of flight to site not considered. Private land not considered due to long term feasibility. Base map and data from OpenStreetMap and OpenStreetMap Foundation.

**Fig 6:**
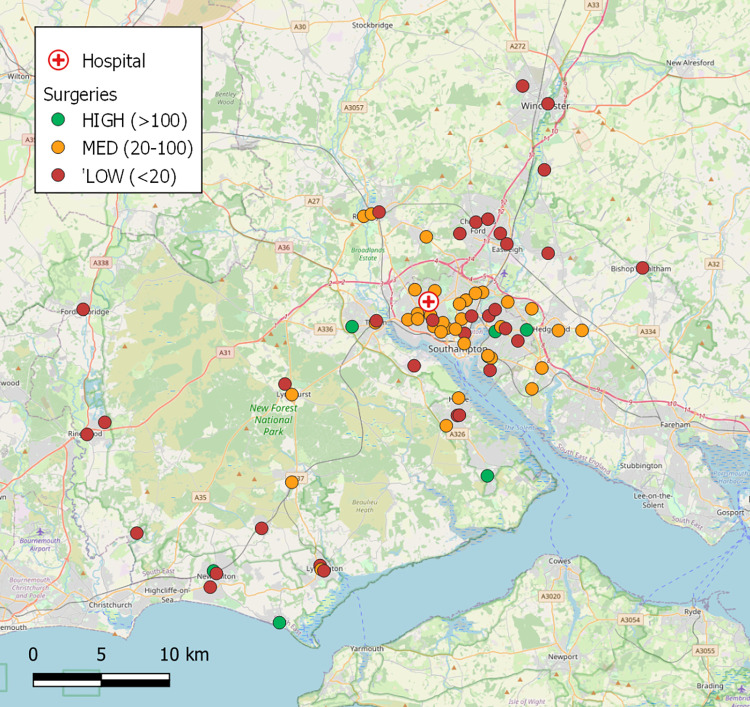
Surgery locations–coloured by daily sample production rate. Base map and data from OpenStreetMap and OpenStreetMap Foundation.

**Fig 7:**
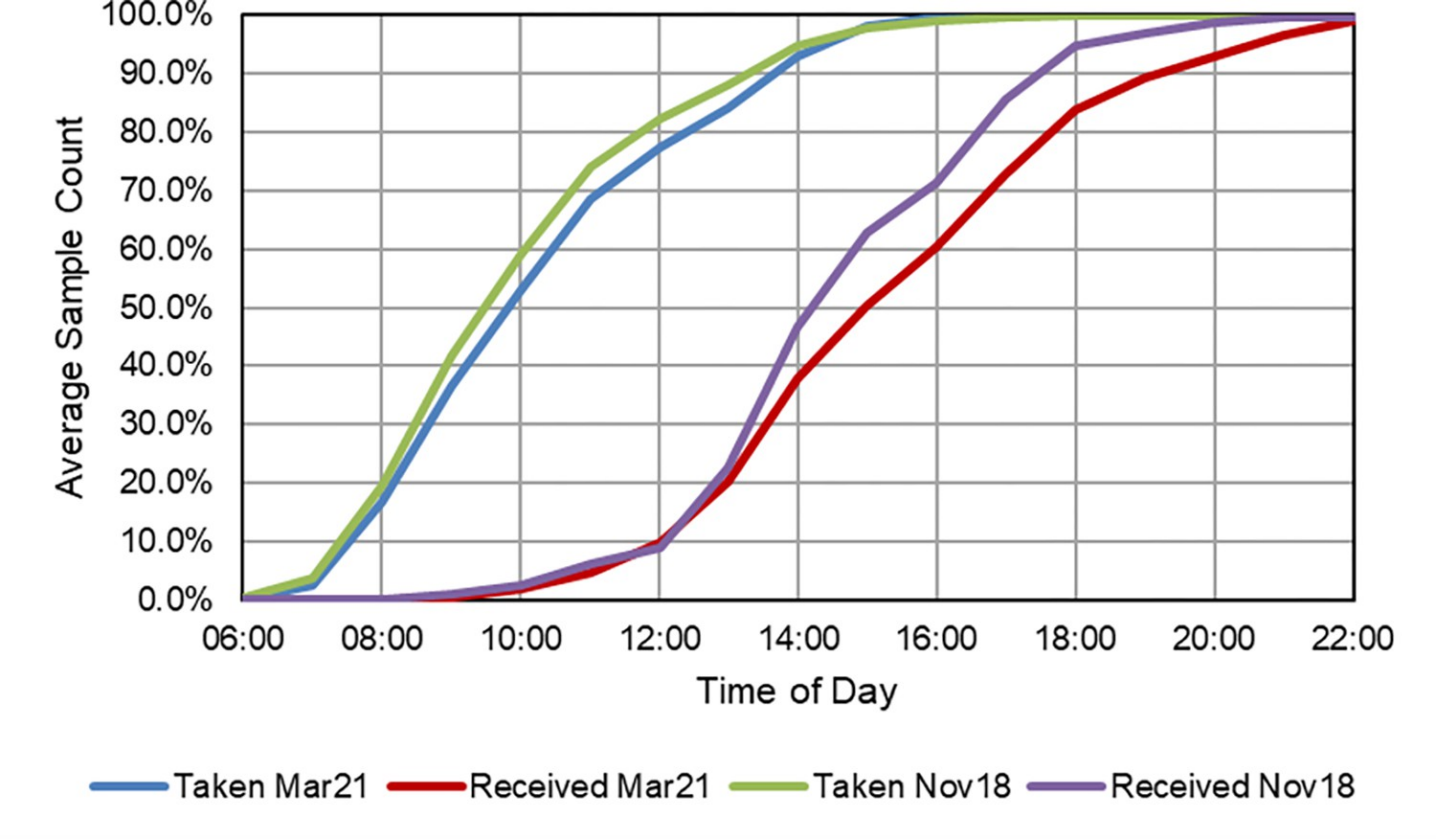
Plot of GP sample records for Southampton—"Bleed Time" (Time Taken) vs. Time Received at SGH laboratory. March 2021 and November 2018 datasets shown to demonstrate minimal long-term change.

In the case of Southampton, part of the ‘South 6 Pathology Network’ [25], patient samples were collected from 79 GP surgeries on a daily basis (Fig 5) by ten van rounds and transported to SGH for analysis. On a typical day (Monday to Friday), each vehicle (diesel-fuelled Vauxhall Vivaro or Ford Transit, GVW 2.9T, Max. Payload 1.2T, [77]) was making approximately 20 collections from surgeries (310 samples/van) and driving 114 km, taking an average of 4 hours 13 mins. Across the rounds, approximately 3,100 samples were produced on the average weekday, equating to roughly 40 samples per surgery, but considerable differences were observed in the mean sample generation rates by surgery, with 9% producing in excess of 100 samples per day, 47% producing between 20 and 100, and 44% producing under 20 [78] (Fig 6). The rate of sample production was not evenly distributed throughout each day, with a clear peak in patient samples being taken during the mid-morning with maximum production observed between 09:00 and 10:00. The data suggested that 79% of the daily samples taken at GP surgeries had been bled before 12:00, but only 12% of these had reached the pathology lab for analysis by this time (Fig 7). Comparison of sample production between days of the week suggested that Tuesdays had significantly greater numbers of samples produced (23%) compared to 19% on each of the other weekdays (M-F) (X^2^_(3)_ = 19.81, P<0.001).

### Determining GP surgeries appropriate for service via UAV

**Fig 8:**
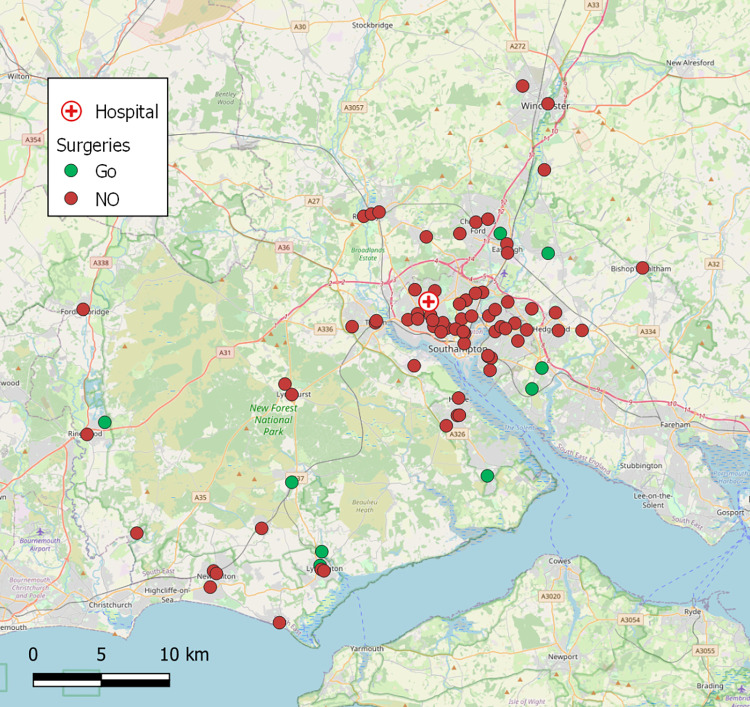
GP Surgeries in the Southampton area coloured by GO/NO-GO Status. GO = suitable for a 5m fixed-wingspan craft to land without excessive ground overflight risk (mean risk of a fatality on the ground due to a UAV crashing along its flightpath = 1**×**10^−7^ or lower). Airspace restrictions are not considered. Base map and data from OpenStreetMap and OpenStreetMap Foundation.

Following an audit of possible landing locations at the 79 GP surgeries in the Southampton area, it was found that 20% of sites could theoretically support landing a VTOL fixed-wing platform with a 5m wingspan UAV, capable of carrying a medium sized Versapak, at a practical distance from surgeries, (Fig 8). This audit involved visually inspecting each surgery via Google Earth and quantifying the size of nearby areas that could be suitable for landing without significantly affecting the existing purpose (e.g., a public park should still be able to function as one, even after cordoning a landing site), subject to landowners’ permission. The results suggested that 49% of surgeries were suitable for smaller VTOL-only platforms (assumed a UAV footprint of 2m). Under UK aviation regulations, set out by the Civil Aviation Authority (CAA), UAVs must be 50m from people or property not in the operator’s control, though reasonable exceptions are negotiable, which would be required in some circumstances [64,65]. Other mechanisms, such as cables and parachutes, can enable collection without landing, though CAA regulations explicitly state that items should not be dropped from UAVs during flight [79], whilst underslung loads may also limit aerodynamic performance, increasing energy use and limiting range.

Further to the practicality of landing locations is the flight path which should be followed. Flight paths must account for a variety of factors, such as other airspace restrictions (e.g., airport control zones) to ensure safety for other users, overflight ground risk (e.g., flying over populous areas may be riskier in the event of crash landing), nature reserves, and noise. These considerations can be addressed using planning tools and UAV Traffic Management (UTM) systems, though if a surgery is located in an area which is not accessible without infringing regulations or bylaws [80], it may not be possible to serve it. As a result, the number of possible surgeries which can be served at present is likely to be more limited.

To enable UAV service at some of these otherwise unsuitable GP surgery sites, transfer to an appropriate collection site by land logistics may be possible. This would maintain some of the environmental and speed benefits of UAVs, but potentially inflate the costs of operation. Additionally, contracting and coordinating the multi-leg journeys presents new challenges in terms of optimisation, with secure handovers and well aligned timings being critical for such a model to succeed.

**Fig 9:**
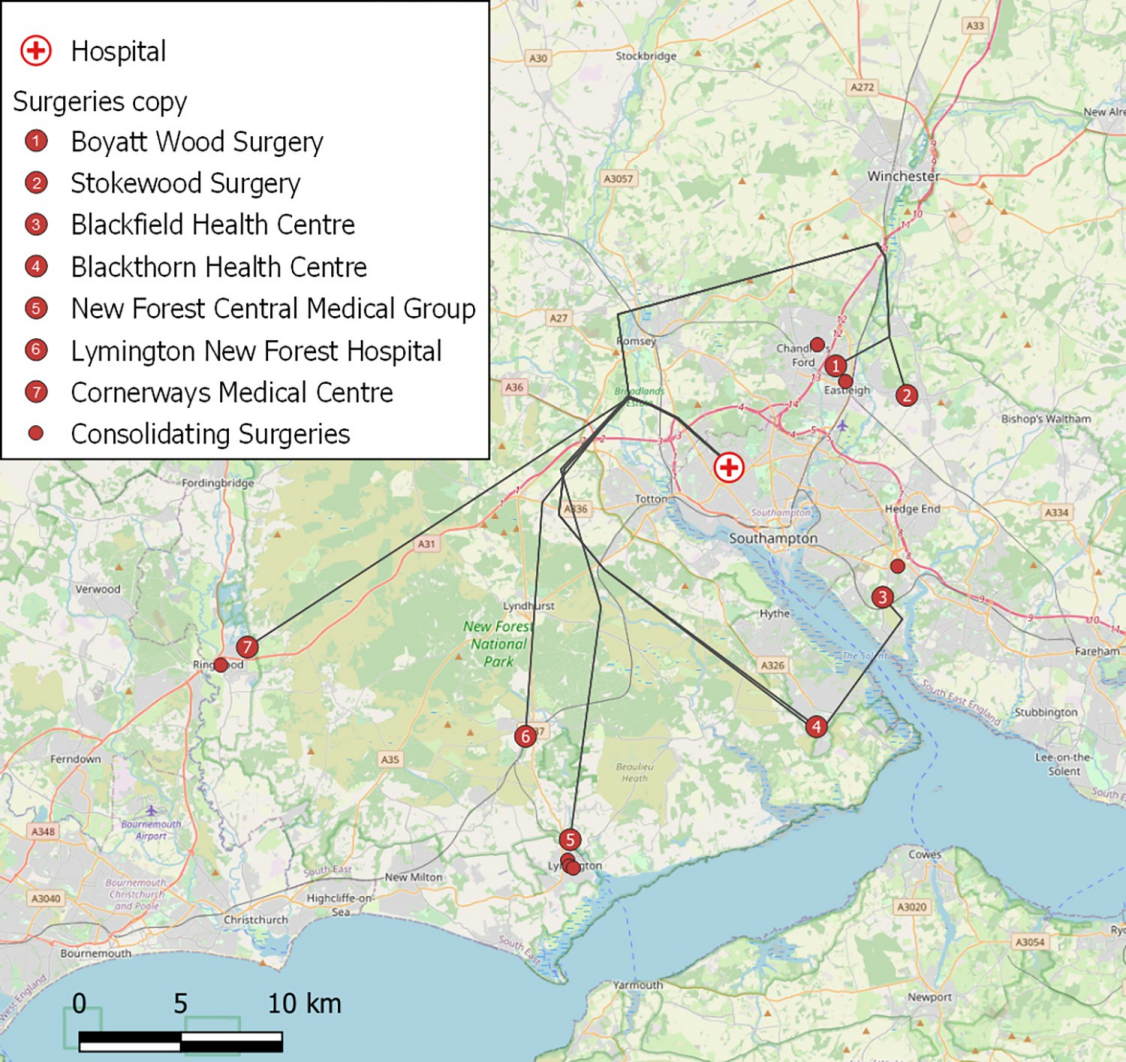
Locations of Doctors’ surgeries included in the investigation. Grey lines indicate UAV flightpaths. Base map and data from OpenStreetMap and OpenStreetMap Foundation.

Based on the given assumptions regarding landing space, ground risk (Fig 8), and airspace restrictions around Southampton Airport, nine surgeries were identified for UAV service (out of the 79 in/around Southampton in total). Two of these nine were within the catchment areas of neighbouring surgeries also classified as suitable, and therefore assumed to be serviced as satellite surgeries by UAVs landing at the nearby primary surgery, resulting in seven primary surgeries being used in the analysis. The planned flight path routes from SGH to these seven surgeries are shown in Fig 9, using a shared path over the urban area around the hospital before splitting in different directions. Seven additional surgeries (i.e., satellite surgeries, including the two classified as suitable) were included in the investigation (smaller points in Fig 9) through being within the catchment areas of the seven primary surgeries.

### Characteristics of a UAV collection service

Results suggested that 13% of the ~70,000 samples generated across the area in March 2021 (9,298–27 generated at weekends = 9,271) had the potential to be served by a UAV collection service using multiple Mugin-5 Pro UAVs involving 194 return flights and 46 taxi trips (i.e., 46 flights which had to be replaced by taxis due to poor weather) at a total cost of £13,633. This equates to average weekly values of 1,628 flown samples/week (387 by contingency taxi), 42 flights/week (10 contingency taxis) at a cost of £2,964/week (Table 2). These cost estimates were based on a foreseeable future situation where automated UAV operations have been established, allowing a mission commander to manage multiple UAVs simultaneously (two per operator), removing the need for safety pilots for take-off and landing [81]. The number of UAVs required for long-term operations has not been identified as this would depend on the demand faced and the wider UAV and air operations in the area.

A further analysis of potential costs based on present day (2021) personnel requirements in the UK, where commercial BVLOS UAV operations are still in their infancy (i.e., one mission commander per UAV and safety pilots for take-off and landing), resulted in much higher costs for a UAV collection service (£8,676 /week) shown by Cost 2 in Table 1 (with personnel making up 86% of the costs, reducing to 56% in the future situation).

**Table 1:** Summary of flights, samples and costs for the UAV collection service.

Statistic		B’field	B’thorn	Boyatt Wood	S’wood	C’ways	New F’st Central	Lym. Hosp.	All Surgeries
**Monthly** **Totals**	Flights	32	40	4	3	1	19	95	194
	Flown Samples	1,060	1,589	10	8	1	519	4,302	7,489
	Taxis	3	10	1	1	1	4	26	46
	Samples by taxi	105	392	2	3	1	67	1,212	1,782
	Cost 1	£2,034	£3,189	£269	£209	£101	£1,108	£6,724	£13,633
	Cost 2	£6,218	£9,651	£813	£618	£228	£3,239	£19,146	£39,912
**Daily** **Avg.**	Flights	1.4	1.7	0.2	0.1	0.0	0.8	4.1	8.4
	Flown Samples	46.1	69.1	0.4	0.3	0.0	22.6	187.0	325.6
	Taxis	0.1	0.4	0.0	0.0	0.0	0.2	1.1	2.0
	Samples by taxi	4.6	17.0	0.1	0.1	0.0	2.9	52.7	77.5
	Cost 1	£88	£139	£12	£9	£4	£48	£292	£593
	Cost 2	£270	£420	£35	£27	£10	£141	£832	£1,735
**Weekly** **Avg.**	Flights	7.0	8.7	0.9	0.7	0.2	4.1	20.7	42.2
	Flown Samples	230.4	345.4	2.2	1.7	0.2	112.8	935.2	1628.0
	Taxis	0.7	2.2	0.2	0.2	0.2	0.9	5.7	10.0
	Samples by taxi	22.8	85.2	0.4	0.7	0.2	14.6	263.5	387.4
	Cost 1	£442	£693	£58	£46	£22	£241	£1,462	£2,964
	Cost 2	£1,352	£2,098	£177	£134	£50	£704	£4,162	£8,676

Cost 1 is for the reasonably foreseeable future situation where multiple UAVs are overseen by one ground controller; Cost 2 is for the current situation involving safety pilots.

**Fig 10:**
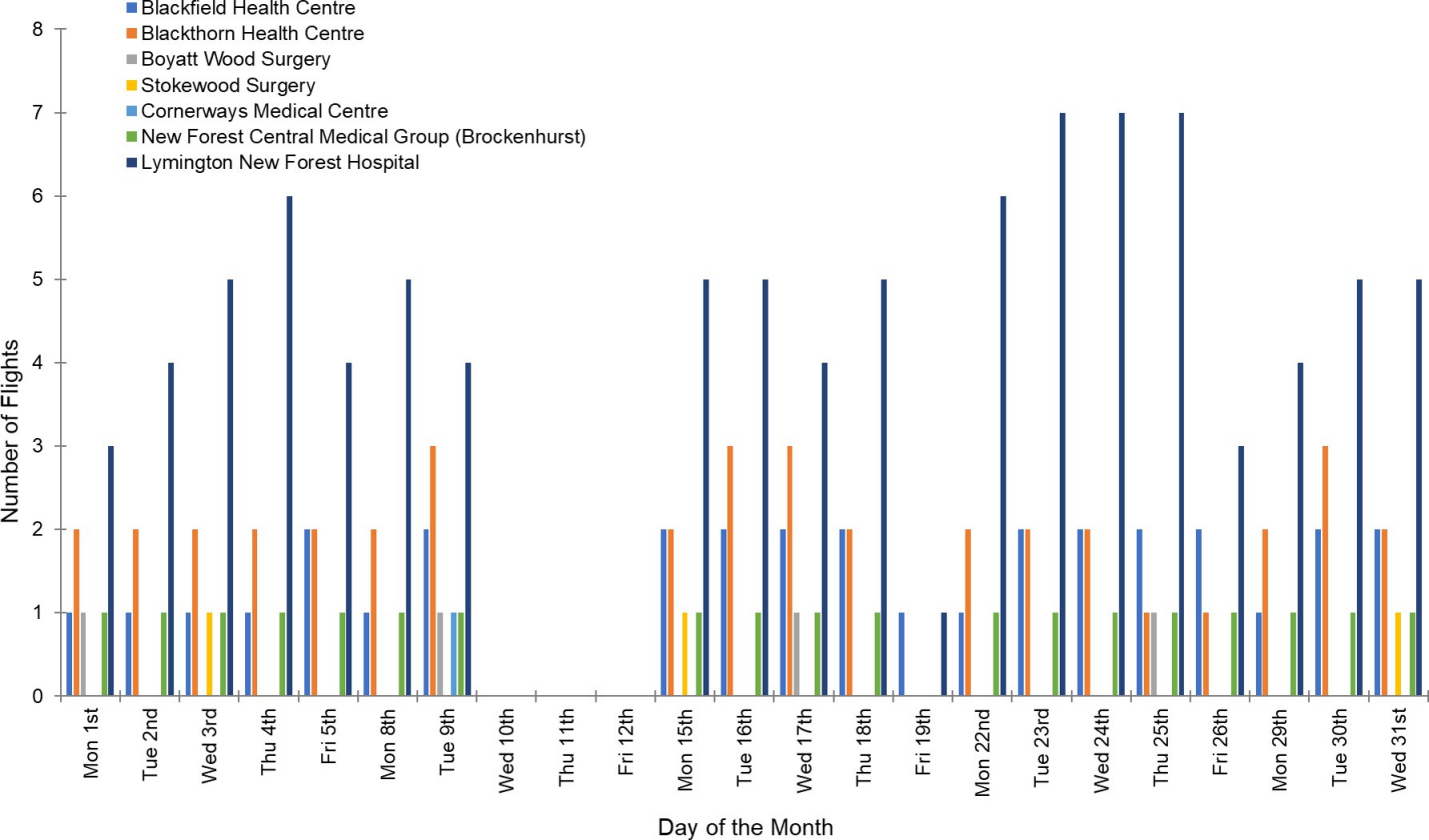
Number of flights per day for the UAV collection service during March 2021.

An analysis of historical weather data for the flight routes in question suggested that of an original 240 monthly flights, 46 (19%) would not be possible under the weather conditions experienced at either the hospital, surgery or both locations. The modelled alternative (a direct taxi service to the hospital) would result in performance reductions (slower delivery times) and potential additions to tailpipe emissions (dependent on the type of taxi modelled), however, they did introduce cost savings over the planned UAV service (e.g., Blackfield surgery return flight = £60 vs. taxi one-way = £33.11) which calls into question the financial value of a UAV service in this case. The weather data also suggested that the distribution of cancelled UAV flights was not even, with only a few days seeing the majority of cancellations (10^th^-12^th^ March 2021 in Fig 10). The wind tolerance of the Mugin-5 Pro UAV used in this desktop analysis was assumed to be 10 m/s (19.4 knots); a level that is less than the weather-resistant UAVs described by Gao et al. [38] (27 knots, 14 m/s). This further emphasises the findings of Koshta et al. [33], suggesting that UAV weather-proofing needs to improve to guarantee reliable service.

Table 2 shows an example of a typical daily flight schedule (Friday 19^th^ March 2021), indicating how some surgeries generate far more samples and therefore UAV flights compared to others which was a consistent observation throughout the month, as shown in the chart of the daily number of flights generated by each surgery (Fig 10).

**Table 2:** Example daily flight schedule for the UAV collection service.

B’field Time (Smp.) Cost 1 Cost 2	B’thorn Time (Smp.) Cost 1 Cost 2	Boyatt Wood Time (Smp.) Cost 1 Cost 2	S’wood Time (Smp.) Cost 1 Cost 2	C’ways Time (Smp.) Cost 1 Cost 2	New F’st C’tral Time (Smp.) Cost 1 Cost 2	Lym. Hosp. Time (Smp.) Cost 1 Cost 2	All Surgeries Total
						09:10 (50) £60 £191	
	10:30 (50) £75 £236						
						11:45 (50) £60 £191	
						14:20 (50) £60 £191	
	14:45 (47) £75 £236						
15:05 (50) £60 £191							
					15:20 (26) £52 £164		
16:25 (5) £60 £191							
						17:30 (13) £60 £191	
**Totals**	Flts. (Smp.) Cost 1 Cost 2						
2 (55) £121 £382	2 (97) £149 £472	0 (0) £0 £0	0 (0) £0 £0	0 (0) £0 £0	1 (26) £52 £164	4 (163) £242 £765	12 (440) £746 £2,359

Time is UAV departure time; Smp. is number of samples on-board; Cost 1 is for the reasonably foreseeable future situation where multiple UAVs are overseen by one ground controller; Cost 2 is for the current situation involving safety pilots. Shaded cells indicate UAV flights.

### Comparison of BAU and intervention scenarios

The results (Table 3) suggested that replacing two vans with multiple UAVs serving 14 different surgeries (7 primary, direct by UAV; and 7 satellite, by connecting ground cycle transportation) would reduce overall BAU van round time by 17.6% (37:05 hours) and distance travelled by 23.0% (1,306 vehicle-kilometres; vkm) per week. In terms of emissions benefits, the daily removal of two diesel vans would reduce tailpipe CO_2_ emissions by 23.0% (365 kg) per week but the use of contingency taxis to cover the instances where flights could not take place due to adverse weather adds a further 49kg of CO_2_ each week, resulting in a net emissions reduction of 19.9% (317kg).

**Table 3:** Weekly road vehicle total costs, round times, van vkm, and CO_2_ emissions (inc. contingency taxis).

Scenario	Number of Rounds/Day	Van Costs (£)	Van Round Time (h:m)	Van Vkm (km)	CO_2_ (kg)
BAU	10	£3,911	210:50	5,686	1,589
Intervention	8	£3,135	173:45	4,380	1,272
Reduction	2	£776 (+£196 MECs)	37:05	1,306	317

Reduction costs show Marginal External Cost reductions in brackets.

Offsetting the cost reduction resulting from fewer van collections (-£776/week, Table 3) against the cost of introducing multiple UAVs (£2,964/week, Table 1) produces a net cost increase of (£2,964-£776 =) £2,188/week for UAVs to service the 14 surgeries; approximately a £59 increase per hour of van driving time saved. In terms of the overall cost for providing a sample collection service to all 79 surgeries, this represents a 56% increase from the BAU scenario which costs £3,911/week based on van-only collections (Table 3) to the intervention scenario costing £6,099/week based on a combination of collection by UAV (and taxi) (£2,964/week in Table 1) and van (£3,135/week in Table 3). It should be noted that UAV costs in this analysis do not match those given by Jenkins et al. (2017), particularly in terms of the cost of labour and the platform itself [39]. This deviation is, in part, due to the larger UAV being used, and the assumptions made around UAV management by staff.

In comparison to existing collections by van, a UAV collection service for the 14 surgeries featured in this investigation represents a considerable increase in cost (i.e., £2,964/week for UAVs *cf*. £776/week for vans) for a relatively small gain in terms of reduced transit time (37 hours/week). The results (Table 1) suggested that a UAV integrated collection service is less cost effective for some surgeries because they generate far fewer samples than others, resulting in inefficient transport with less than full payloads, although ultimately, all samples must be collected.

For example, values for average payload and cost per sample (including collections by replacement taxi when necessary) range from: Lymington New Forest Hospital with (5,514/121 =) 45.6 samples/collection and (£6,792/5,514 =) £1.23/sample, respectively; to Cornerways Medical Centre with (2/2 =) 1 sample/collection and (£117/2 =) £58.50/sample, respectively. This disparity in cost effectiveness between surgeries suggested that, when further optimisation of the collection service is conducted, some form of mixed-mode (i.e., van-UAV) hybrid collection service involving localised consolidation of samples by vans performing routine timetabled collections for onward transport of consolidated payloads by UAV might represent a less expensive solution. For example, UAV flights to the relatively inefficient surgeries (i.e., low samples/flight and high costs/sample) at New Forest Central Medical Group and Cornerways Medical Centre (surgeries 6 and 7 in Fig 9, respectively) could be eliminated using scheduled van collections instead, consolidating samples at the nearby (and already efficient) Lymington New Forest Hospital (Surgery 5 in Fig 9) for onward transport to SGH.

Investigating the Marginal External Costs (MECs, often referred to as society costs) associated with the reduction in road vehicle mileage can demonstrate some further benefits of the UAV service. Accounting for greenhouse gas (GHG) emissions, congestion, and air pollution costs, the intervention presented reduces the MECs of the system by £196 per week (GHG = £81 reduction, congestion = £116 reduction, air pollution–NO_x_ <£1 reduction, PM_2.5_ = <£1 reduction) [74–76]. Whilst these cost reductions will not directly benefit the operators, they highlight the potential benefits of UAVs more widely as they may help the health service operate more efficiently, though quantifying this is considerably more difficult.

An advantage of a UAV collection service is more frequent and expeditious (i.e., shorter travel times) collections and deliveries compared to the existing van collection service, particularly if a mixed-mode consolidation approach were to be utilised because UAVs would reach full capacity (i.e., 50 samples) in shorter timescales and be ready to depart from surgeries earlier in the day. The existing van collection service for all 79 surgeries in/around Southampton involves 10 collection-rounds/day in BAU (Table 3), with each round including between one and three delivery stops at the SGH pathology laboratory, generating 21 deliveries/day, generally concentrated during the period from mid-morning to early afternoon. In contrast, the UAV collection service for just the 14 surgeries investigated generates an average of 10.4 deliveries/day (Table 1), with deliveries spread throughout the day from ~09:00 to ~17:00 (Table 2).

Improvements in journey times were made in all cases where UAVs were used, with the original van travel times ranging from 20 to 265 minutes (from the investigated surgeries to SGH) with an average of 88 minutes, whilst UAV flight times ranged from 19 to 30 minutes with an average of 24 minutes. The contingency taxi services range from 24–60 minutes, meaning any cancelled flights would experience much slower delivery. These figures correlate with the similar study by Comtet et al. [32] who also noted large benefits in journey times but suggested there may be concerns over a need for a backup system.

A more frequent and expeditious service has the potential to provide three benefits: (i) better workflow management and control for laboratory staff and/or equipment of samples arriving for analysis; (ii) samples spend less time subjected to uncontrolled in-vehicle conditions, which may be harmful to samples (e.g., excessive temperature or vibration conditions) and affect the integrity of analysis results [52,82,83]; and (iii) reduced bleed-to-diagnosis times, which would be advantageous for any time-critical samples. Blood samples taken from cancer patients to inform the prescription of chemotherapy treatments, a significant proportion of which are bespoke manufactured for their specific needs a few hours prior to administration are a good example. Discussions with NHS practitioners has suggested that there may be further advantages from separating some samples for fast-track analysis, and that smaller UAVs could enhance current collection services in this regard, improving the efficiency of collection services and ultimately patient treatment plans.

In this regard, further research is needed to investigate (i) the life-enhancing benefits to patients from fast-track sample analysis using UAVs and the cost-benefit trade-off to the NHS of using such services; and (ii) the potential productivity improvements that could be gained by the analysis laboratories from having more evenly distributed sample delivery patterns. The benefits could be monetarised based on the Quality Adjusted Life Year (QALY), a metric commonly used for the economic evaluation of medical interventions [84].

## Conclusions

Trials of UAVs for medical logistics UAV are becoming more prevalent throughout the world although few have translated into fully commercial services to date. This research used a series of real-world datasets to investigate the practicality, benefits, and costs of integrating UAVs into an existing NHS diagnostic specimen collection service operating in the Solent region of the UK. An audit of the existing van-based collection activity highlighted the desire for earlier and more frequent delivery of samples to the analysis laboratory, shorter transit durations, and faster turn round times from bleed-to-diagnosis.

Keeping with the current requirement to use industry approved packaging (a medium Versapak carrier, (460mm (w), 255mm (D), 305mm (H))) to transport samples from surgeries, a 5m wingspan VTOL UAV was modelled as the platform necessary to transport such a unit load from suitable GP surgeries with the appropriate UN3373 dangerous goods certification. Given the scale of the UAV required, only 20% of the GP surgeries investigated would be practically capable of receiving such a platform in terms of safe landing space, and after further consideration of immediate ground risk, only 11% were deemed suitable.

In an exploratory desktop analysis, 14 out of 79 surgeries could be served by UAV: seven directly, and seven through ground-based logistics transfers. The results suggested that an average of 1,628 samples could be served by UAV each week, resulting in 42 flights/week with 10 taxi services to cover periods where weather limited flying. This resulted in an approximate total service cost of £2,964/week if regulations developed to relax UAV personnel constraints. Whilst this created a 20% reduction in tailpipe CO_2_ emissions (excl. taxis) and van logistics costs, the overall service cost increased by 56%, making any long-term UAV service financially challenging. Some sites were more cost effective than others, with per-sample operating costs ranging from £1.23 to £58.50; however, the business-as-usual methods remained more cost-effective in all cases. Airspace management and dangerous goods training costs were not considered in the analysis and would create further costs if the system was implemented.

Introducing UAVs does offer other potential benefits such as faster diagnosis and subsequent patient treatment plan development due to the reduced travel times, although quantifying their true value to the health service is more challenging. The introduction of UAVs in this case study reduced the marginal external costs (greenhouse gas emissions, congestion, and air pollution) by £196 per week and cut travel times to UAV served sites by 72% when weather conditions were suitable.

A previous investigation into the practicality of UAV delivery by McKinnon [85] highlighted that widespread commercial operations were unlikely, due to the prohibitive operating costs relative to the added value generated by services. In the context of patient diagnostics collections in the case study area, the journey time benefits that could be gained from the introduction of UAVs would come at a significant cost compared to business-as-usual van-based logistics, unless a tangible benefit to patient care can result. Achieving operations based on significant subsidies, like Zipline in Ghana [4], is not sustainable.

Should changes to regulations enable load consolidation or enable UAVs to service a greater number of sites, there may be a point at which UAVs can more effectively integrate into existing fleets and eliminate the need for one or more vans to visit the more remote surgeries, although it would be advisable to fully evaluate the costs and benefits of the system should this occur. This study was limited in that it considered UAVs and vans in fixed and isolated rounds, with no allowance for overlap. Furthermore, whilst the UAV modelled was suitable for carrying the Versapak medical carrier, sites with lower demand may have the potential to be served by a smaller UAV at lower cost and with greater flexibility.

## Supporting information

S1 Table Cost assumptions used in the desktop analysis. Costs and life expectancy based on Cascade UAV research consortium development experience (www.cascadeuav.com). Example components are shown to demonstrate possible options, the craft described in this paper was not necessarily fitted with these exact components [72,86–91].
(DOCX)

## References

[1] Rosser JC, Vignesh V, Terwilliger BA, Parker BC. Surgical and Medical Applications of Drones: A Comprehensive Review. JSLS. 2018;22. doi: 10.4293/JSLS.2018.00018 30356360 PMC6174005

[2] UPDWG. Medical Drone Delivery Database (MD3). In: UPDWG [Internet]. 2022 [cited 4 Jan 2022]. Available: https://www.updwg.org/md3/.

[3] Mendelow B, Robertson J, Muir P, Boshielo BT. Development of e-Juba, a preliminary proof of concept unmanned aerial vehicle designed to facilitate the transportation of microbiological test samples from remote rural clinics to National Health Laboratory Service laboratories. South African Medical Journal. 2007;97: 1215–1218. 18250941

[4] Ackerman E, Koziol M. In the Air With Zipline’s Medical Delivery Drones. In: IEEE Spectrum [Internet]. 30 Apr 2019 [cited 4 Jan 2022]. Available: https://spectrum.ieee.org/in-the-air-with-ziplines-medical-delivery-drones.

[5] McNabb M. Zipline in Nigeria: Cross River State Partners for Medical Drone Delivery of Vaccines, Medicine, and Blood Supplies. In: DRONELIFE [Internet]. 10 May 2021 [cited 4 Jan 2022]. Available: https://dronelife.com/2021/05/10/zipline-in-nigeria-cross-river-state-partners-for-medical-drone-delivery-of-vaccines-medicine-and-blood-supplies/.

[6] NHS. Delivering a ‘Net Zero’ National Health Service. Oct 2020 [cited 14 Apr 2021]. Available: https://www.england.nhs.uk/greenernhs/wp-content/uploads/sites/51/2020/10/delivering-a-net-zero-national-health-service.pdf.

[7] Médecins Sans Frontières. Innovating to reach remote TB patients and improve access to treatment. 2014 [cited 31 Jan 2022]. Available: https://www.msf.org/papua-new-guinea-innovating-reach-remote-tb-patients-and-improve-access-treatment.

[8] Africa Drone Forum. Home. In: African Drone Forum [Internet]. 2022 [cited 31 Jan 2022]. Available: https://www.africandroneforum.org/.

[9] Grote M, Pilko A, Scanlan J, Cherrett T, Dickinson J, Smith A, et al. Pathways to Unsegregated Sharing of Airspace: Views of the Uncrewed Aerial Vehicle (UAV) Industry. Drones. 2021;5: 150. doi: 10.3390/drones5040150

[10] Kesteloo H. Matternet drones to deliver COVID-19 tests in Berlin. In: DroneXL.co [Internet]. 25 Nov 2020 [cited 27 Jan 2021]. Available: https://dronexl.co/2020/11/25/matternet-drones-to-deliver-covid-19-tests-berlin/.

[11] Hern A. DHL launches first commercial drone “parcelcopter” delivery service. In: the Guardian [Internet]. 25 Sep 2014 [cited 1 Oct 2020]. Available: http://www.theguardian.com/technology/2014/sep/25/german-dhl-launches-first-commercial-drone-delivery-service.

[12] sUAS News. Skyports partners with Swoop Aero to provide UK-wide drone delivery service. In: sUAS News—The Business of Drones [Internet]. 21 Jan 2021 [cited 27 Apr 2021]. Available: https://www.suasnews.com/2021/01/skyports-partners-with-swoop-aero-to-provide-uk-wide-drone-delivery-service/.

[13] Smith P. Drones used to deliver Covid tests to Scotland’s rural communities. In: ITV News [Internet]. 17 Mar 2021 [cited 27 Apr 2021]. Available: https://www.itv.com/news/2021-03-17/drones-used-to-deliver-covid-tests-and-samples-to-scotlands-rural-communities.

[14] Kersley A. The slow collapse of Amazon’s drone delivery dream. Wired UK. 2021. Available: https://www.wired.co.uk/article/amazon-drone-delivery-prime-air. Accessed 31 Jan 2022.

[15] Banks T, Wyrobek K. The designer who built a drone to save lives. In: Design Week [Internet]. 31 Oct 2019 [cited 9 Nov 2021]. Available: https://www.designweek.co.uk/issues/28-october-3-november-2019/designer-drone-zipline-rwanda-keenan-wyrobek/.

[16] Levin A. Alphabet’s (GOOG) Drone Unit Tests Deliveries From Mall Roof—Bloomberg. 6 Oct 2021. Available: https://www.bloomberg.com/news/articles/2021-10-06/mall-retailers-get-boost-from-alphabet-drones-delivering-sushi. Accessed 9 Nov 2021.

[17] Boon MA, Drijfhout AP, Tesfamichael S. Comparison of a Fixed-Wing and Multi-Rotor Uav for Environmental Mapping Applications: a Case Study. ISPRS—International Archives of the Photogrammetry, Remote Sensing and Spatial Information Sciences. 2017;42W6: 47–54. doi: 10.5194/isprs-archives-XLII-2-W6-47-2017

[18] Collier AH. Drones to the Rescue! How the Public Benefit of Drones Far Outweighs the Risks | Commercial UAV News. 17 Mar 2020 [cited 30 Sep 2020]. Available: https://www.commercialuavnews.com/public-safety/drones-to-the-rescue-how-the-public-benefit-of-drones-far-outweighs-the-risks.

[19] Ilancheran M. COVID-19, Medical Drones, & The Last Mile Of The Pharma Supply Chain. 2020 [cited 15 Jan 2021]. Available: https://www.pharmaceuticalonline.com/doc/covid-medical-drones-the-last-mile-of-the-pharma-supply-chain-0001.

[20] Walcutt L. Zipline Is Launching The World’s Largest Drone Delivery Network In Tanzania. In: Forbes [Internet]. 24 Aug 2017 [cited 30 Sep 2020]. Available: https://www.forbes.com/sites/leifwalcutt/2017/08/24/zipline-is-launching-the-worlds-largest-drone-delivery-network-in-tanzania/.

[21] DPA. Zipline launches fastest delivery drone in the world. 18 Jun 2018 [cited 28 Sep 2020]. Available: http://www.dpaonthenet.net/article/155369/Zipline-launches-fastest-delivery-drone-in-the-world.aspx.

[22] Nisingizwe MP, Law M, Bimpe I. Effects of drone delivery on the blood product supply chain in Rwanda. 2022 May 19; Online—Hosted by UPDWG.

[23] Guy’s and St. Thomas’ NHSFT. Pathology services: Viapath. 2019 [cited 2 Jan 2020]. Available: https://www.guysandstthomas.nhs.uk/Home.aspx.

[24] NHS, Sedman R. Pathology Specimen Transport. 2020. Available: https://www.cddft.nhs.uk/media/615106/transport%20sop.pdf.

[25] NHS. Pathology networks | NHS Improvement. 8 Sep 2017 [cited 22 Nov 2019]. Available: https://web.archive.org/web/20200808010235/https://improvement.nhs.uk/resources/pathology-networks/.

[26] Dobbin J, Cotton S, Wilding R. True Blood: challenges of the bloodsupply chain in England. CILT Focus. Nov 2009. Available: http://www.richardwilding.info/uploads/7/2/0/3/7203177/true_blood_-_logistics_focus_november_2009_-_dobbinwildingcotton.pdf. Accessed 26 Nov 2019.

[27] NHS. Invitation to Tender to Supply the Pathology Collection Network for The East and South East London NHS Pathology Partnership. 2021.

[28] Cherrett T, Moore A. Saving the NHS: A case study evaluation of drones and cargo cycles for surgery-to-hospital pathology logistics in Southampton UK. Transportation Research Board (TRB) 99th Annual Meeting. 12–16 January, Washington D.C.; 2020.

[29] Allan R. Southampton Pathology Operations University Project Meeting. 2019.

[30] Ford M. Covid-19 testing “much slower” in community, says Welsh RCN. In: Nursing Times [Internet]. 24 Jul 2020 [cited 29 Jan 2021]. Available: https://www.nursingtimes.net/news/coronavirus/covid-19-testing-much-slower-in-community-says-welsh-rcn-24-07-2020/.

[31] Skyports. “Call the drone”—delivering for the NHS. In: Skyports [Internet]. 12 May 2021 [cited 26 Jul 2021]. Available: https://skyports.net/2021/05/call-the-drone-delivering-for-the-nhs/.

[32] Comtet HE, Keitsch M, Johannessen K-A. Realities of Using Drones to Transport Laboratory Samples: Insights from Attended Routes in a Mixed-Methods Study. JMDH. 2022;15: 1871–1885. doi: 10.2147/JMDH.S371957 36068877 PMC9441146

[33] Koshta N, Devi Y, Chauhan C. Evaluating Barriers to the Adoption of Delivery Drones in Rural Healthcare Supply Chains: Preparing the Healthcare System for the Future. IEEE Transactions on Engineering Management. 2022; 1–13. doi: 10.1109/TEM.2022.3210121

[34] Lucas Austin P. Amazon Drone Delivery Was Supposed to Start By 2018. Here’s What Happened Instead. Time. 2 Nov 2021. Available: https://time.com/6093371/amazon-drone-delivery-service/. Accessed 31 Jan 2022.

[35] Stolaroff JK, Samaras C, O’Neill ER, Lubers A, Mitchell AS, Ceperley D. Energy use and life cycle greenhouse gas emissions of drones for commercial package delivery. Nature Communications. 2018;9: 409. doi: 10.1038/s41467-017-02411-5 29440638 PMC5811440

[36] Daleo J. DHL pulling its Parcelcopter drone, ceasing drone development. FreightWaves. 9 Aug 2021. Available: https://www.freightwaves.com/news/dhl-pulling-its-parcelcopter-drone-ceasing-drone-development. Accessed 31 Jan 2022.

[37] Drone Delivery Was Supposed to be the Future. What Went Wrong?—YouTube. 2022. Available: https://www.youtube.com/watch?v=J-M98KLgaUU&ab_channel=WendoverProductions.

[38] Gao M, Hugenholtz CH, Fox TA, Kucharczyk M, Barchyn TE, Nesbit PR. Weather constraints on global drone flyability. Sci Rep. 2021;11: 12092. doi: 10.1038/s41598-021-91325-w 34103585 PMC8187708

[39] Jenkins D, Vasigh B, Oster C, Larsen T. Forecast of the Commercial UAS Package Delivery Market. 2017 May. Available: https://web.archive.org/web/20170601042642/ http:/nebula.wsimg.com/28ad8975cfef999798fa4b20e7238f67?AccessKeyId=02FB2B5A65F7EC056121&disposition=0&alloworigin=1.

[40] Skybrary. Unmanned Aircraft Systems Traffic Management (UTM). In: SKYbrary Aviation Safety [Internet]. 30 May 2021 [cited 13 May 2022]. Available: https://skybrary.aero/articles/unmanned-aircraft-systems-traffic-management-utm.

[41] Bohlig A. UTM Deep Dive: A Multi-Billion Dollar Market You Can’t Ignore. In: Loup [Internet]. 9 Oct 2017 [cited 17 Jan 2022]. Available: https://loupfunds.com/utm-deep-dive-a-multi-billion-dollar-market-you-cant-ignore/.

[42] Met Office. UK storm season 2019/20. In: Met Office [Internet]. 2020 [cited 4 May 2021]. Available: https://www.metoffice.gov.uk/weather/warnings-and-advice/uk-storm-centre/uk-storm-season-2019-20.

[43] Met Office. UK Storm Centre. In: Met Office [Internet]. 2021 [cited 4 May 2021]. Available: https://www.metoffice.gov.uk/weather/warnings-and-advice/uk-storm-centre/index.

[44] Eurocontrol. London Heathrow LHR / EGLL airport information. 2021 [cited 27 Jul 2021]. Available: https://ext.eurocontrol.int/airport_corner_public/EGLL.

[45] NATS. How does strong wind affect Air Traffic Control? In: NATS Blog [Internet]. 18 Jan 2018 [cited 9 Dec 2021]. Available: https://nats.aero/blog/2018/01/strong-wind-affect-air-traffic-control/.

[46] Heathrow Airport Ltd. Climate change adaptation and resilience progress report. 2016 p. 54. Available: https://assets.publishing.service.gov.uk/government/uploads/system/uploads/attachment_data/file/566147/climate-adrep-heathrow.pdf.

[47] Ottersen G, Planque B, Belgrano A, Post E, Reid PC, Stenseth NC. Ecological effects of the North Atlantic Oscillation. Oecologia. 2001;128: 1–14. doi: 10.1007/s004420100655 28547079

[48] EPSRC. Grants on the web—e-Drone. Engineering and Physical Sciences Research Council, Polaris House, North Star Avenue, Swindon, SN2 1ET; 2020 [cited 19 May 2021]. Available: https://gow.epsrc.ukri.org/NGBOViewGrant.aspx?GrantRef=EP/V002619/1.

[49] UK Government. New transport tech to be tested in biggest shake-up of laws in a generation. In: GOV.UK [Internet]. 16 Mar 2020 [cited 19 May 2021]. Available: https://www.gov.uk/government/news/new-transport-tech-to-be-tested-in-biggest-shake-up-of-laws-in-a-generation.

[50] BBC. Coronavirus: Drones to deliver NHS supplies to Isle of Wight. BBC News. 24 Apr 2020. Available: https://www.bbc.co.uk/news/technology-52419705. Accessed 10 Sep 2020.

[51] Peskett J. Windracers UAV completes heavy payload medical delivery run in UK. In: Commercial Drone Professional [Internet]. 21 Dec 2020 [cited 26 Jul 2021]. Available: https://www.commercialdroneprofessional.com/windracers-uav-completes-heavy-payload-medical-delivery-run-in-uk/.

[52] Oakey A, Waters T, Zhu W, Royall PG, Cherrett T, Courtney P, et al. Quantifying the Effects of Vibration on Medicines in Transit Caused by Fixed-Wing and Multi-Copter Drones. Drones. 2021;5. doi: 10.3390/drones5010022

[53] UNECE. UN Recommendations on the Transport of Dangerous Goods—Model Regulations. 2019 [cited 8 Mar 2021]. Available: https://unece.org/rev-21-2019.

[54] Spire Healthcare. Services | Spire Healthcare. 2021 [cited 27 Apr 2021]. Available: https://www.spirehealthcare.com/pathology/services/.

[55] ICAO. ICAO Doc 9284 Technical Instructions for the Safe Transport of Dangerous Goods By Air Ed. 2019.

[56] Grote M, Cherrett T, Oakey A, Royall PG, Whalley S, Dickinson J. How Do Dangerous Goods Regulations Apply to Uncrewed Aerial Vehicles Transporting Medical Cargos? Drones. 2021;5: 38. doi: 10.3390/drones5020038

[57] Medicines and Healthcare products Regulatory Agency. Rules and Guidance for Pharmaceutical Distributors 2017 (The Green Guide). 10th ed. 2017.

[58] UK CAA. CAP2248—Dangerous Goods RPAS Fundamentals. 2021 [cited 9 Dec 2021]. Available: https://publicapps.caa.co.uk/docs/33/Dangerous%20Goods%20RPAS%20Fundamentals%20(CAP2248).pdf.

[59] Amukele TK, Sokoll LJ, Pepper D, Howard DP, Street J. Can Unmanned Aerial Systems (Drones) Be Used for the Routine Transport of Chemistry, Hematology, and Coagulation Laboratory Specimens? PLOS ONE. 2015;10: e0134020. doi: 10.1371/journal.pone.0134020 26222261 PMC4519103

[60] Versapak. Versapak Insulated Pathology Medical Carrier & Seal Bundle. 2019 [cited 3 Jan 2020]. Available: https://www.versapak.co.uk/versapak-insulated-pathology-medical-carrier-seal-bundle.

[61] BBC. Isle of Wight NHS trust trials drones for chemotherapy deliveries. BBC News. 24 Sep 2021. Available: https://www.bbc.com/news/uk-england-hampshire-58672437. Accessed 15 Nov 2021.

[62] Fraqueiro F, Albuquerque P, Gamboa P. A computer application for parametric aircraft design. Open Engineering. 2016;6: 432–440. doi: 10.1515/eng-2016-0067

[63] Barnard Microsystems UAV design guidelines. 2019 [cited 9 Dec 2021]. Available: https://barnardmicrosystems.com/UAV/uav_design/guidelines.html.

[64] UK CAA. THE NEW UAS REGULATIONS–WHAT’S THE DIFFERENCE? CAP2008. 2020. Available: http://publicapps.caa.co.uk/docs/33/CAP2008_EU_Drone_Rules_Factsheet_V7%206.pdf.

[65] UK CAA. Where you can fly drones | UK Civil Aviation Authority. 2021 [cited 27 Jul 2021]. Available: https://register-drones.caa.co.uk/drone-code/where-you-can-fly.

[66] Savage I. Comparing the fatality risks in United States transportation across modes and over time. Research in Transportation Economics. 2013;43: 9–22. doi: 10.1016/j.retrec.2012.12.011

[67] Pilko A, Sóbester A, Scanlan JP, Ferraro M. Spatiotemporal Ground Risk Mapping for Uncrewed Aerial Systems operations. AIAA SCITECH 2022 Forum. American Institute of Aeronautics and Astronautics; 2021. doi: 10.2514/6.2022–1915

[68] Apian Ltd. Solent Trials. 2021 [cited 4 Jan 2022]. Available: https://www.apian.aero/solent.html.

[69] Allen J, Piecyk M, Cherrett T, Juhari MN, McLeod F, Piotrowska M, et al. Understanding the transport and CO2 impacts of on-demand meal deliveries: A London case study. Cities. 2021;108: 102973. doi: 10.1016/j.cities.2020.102973

[70] Meteostat. Hourly Data | Weather Stations | JSON API | Meteostat Developers. 2021 [cited 21 May 2021]. Available: https://dev.meteostat.net/api/stations/hourly.html.

[71] MuginUAV. Mugin UAV–PROFESSIONAL MANUFACTURER OF UAV AIRFRAMES. 2022 [cited 11 Feb 2022]. Available: https://www.muginuav.com/.

[72] FTA. Manager’s Guide to Distribution Costs 2020. 2020.

[73] WestQuay Cars. Southampton Taxi Fare Calculator Instant Quote. In: West Quay Cars [Internet]. 2021 [cited 9 Dec 2021]. Available: https://westquaycars.com/southampton-taxi-fare-calculator/.

[74] Department for Transport. TAG data book. 2021. Available: https://www.gov.uk/government/publications/tag-data-book.

[75] European Environment Agency. EMEP/EEA air pollutant emission inventory guidebook 2019—European Environment Agency - 1.A.3.b.i-iv Road transport 2019. 2022. Available: https://www.eea.europa.eu/publications/emep-eea-guidebook-2019/part-b-sectoral-guidance-chapters/1-energy/1-a-combustion/1-a-3-b-i/view.

[76] UK Government. Greenhouse gas reporting: conversion factors 2021. 2022. Available: https://www.gov.uk/government/publications/greenhouse-gas-reporting-conversion-factors-2021.

[77] Vauxhall. Vivaro 2011 Models Edition 2. 2011. Available: https://www.vauxhallfleet.co.uk/uploads/historical-data/pdf/3637.pdf.

[78] Oakey A. Investigating the Potential For Autonomous and Cycle-Based Freight Systems to Support the National Health Service as Part of a Mixed-Fleet Logistics Operation (Confirmation Thesis). University of Southampton. 2021.

[79] UK CAA. Making every drone flight safe | UK Civil Aviation Authority. 2021 [cited 27 Jul 2021]. Available: https://register-drones.caa.co.uk/drone-code/making-every-flight-safe.

[80] New Forest NPA. Flying drones. In: New Forest National Park Authority [Internet]. 2021 [cited 9 Dec 2021]. Available: https://www.newforestnpa.gov.uk/visiting/visitor-information/flying-drones-in-the-new-forest-national-park/.

[81] UK CAA. CAP722 Unmanned Aircraft System Operations in UK Airspace–Guidance. 2020.

[82] Anaya-Arenas AM, Chabot T, Renaud J, Ruiz A. Biomedical sample transportation in the province of Quebec: a case study. International Journal of Production Research. 2016;54: 602–615. doi: 10.1080/00207543.2015.1018455

[83] Wilson ML. General Principles of Specimen Collection and Transport. Clinical Infectious Diseases. 1996;22: 766–777. doi: 10.1093/clinids/22.5.766 8722929

[84] NICE. How NICE measures value for moneHow NICE measures value for money in relation to public health interventions. Sep 2013 [cited 9 Dec 2021]. Available: https://www.nice.org.uk/Media/Default/guidance/LGB10-Briefing-20150126.pdf.

[85] McKinnon AC. The Possible Impact of 3D Printing and Drones on Last-Mile Logistics: An Exploratory Study. Built Environment. 2016;42: 617–629. doi: 10.2148/benv.42.4.617

[86] Mugin UAV. Mugin-5 Pro 5000mm Super Large VTOL UAV Platform. 2021 [cited 9 Nov 2021]. Available: https://www.muginuav.com/product/mugin-5-pro-5000mm-super-large-vtol-uav-platform/.

[87] Distributed Avionics. Distributed Avionics—Services. 2022 [cited 7 Feb 2022]. Available: https://www.distributed-avionics.com/Services.

[88] Honeywell. Honeywell UAV SATCOM. 2022 [cited 11 Feb 2022]. Available: https://aerospace.honeywell.com/us/en/pages/sff-uav-satcom.

[89] Lin X, Yajnanarayana V, Muruganathan SD, Gao S, Asplund H, Maattanen H-L, et al. The Sky Is Not the Limit: LTE for Unmanned Aerial Vehicles. IEEE Communications Magazine. 2018;56: 204–210. doi: 10.1109/MCOM.2018.1700643

[90] UK Government. Gas and electricity prices in the non-domestic sector. In: GOV.UK [Internet]. 2021 [cited 4 Jan 2022]. Available: https://www.gov.uk/government/statistical-data-sets/gas-and-electricity-prices-in-the-non-domestic-sector.

[91] NHS. Porter. In: Health Careers [Internet]. 7 Apr 2015 [cited 9 Dec 2021]. Available: https://www.healthcareers.nhs.uk/explore-roles/wider-healthcare-team/roles-wider-healthcare-team/support-services/porter.

