# Supplementary material for: Integrating drones into NHS patient diagnostic logistics systems: Flight or fantasy?
Source: PLoS One. 2022 Dec 22;17(12):e0264669. doi: 10.1371/journal.pone.0264669 (PMC9778562; doi:10.1371/journal.pone.0264669)
Supplement: S1 Table — Costs and life expectancy based on Cascade UAV research consortium development experience (www.cascadeuav.com). Example components are shown to demonstrate possible options, the craft described in this paper was not necessarily fitted with these exact components [72,86–91]. (DOCX) [file pone.0264669.s001.docx]

S1 Table. Cost assumptions used in the desktop analysis. Costs and life expectancy based on Cascade UAV research consortium development experience ([www.cascadeuav.com](http://www.cascadeuav.com)). Example components are shown to demonstrate possible options, the craft described in this paper was not necessarily fitted with these exact components.

| **Item (Qty)** | **Assumed Life (flight-hours)** | **Cost Per Flight-hour** |
| --- | --- | --- |
| Forward Motor (e.g. Dualsky XM6360EA-19 220KV [86]) | 1,000 | £0.10 |
| Forward Electronic Speed Controller (ESC) (e.g. Hobbywing 100A 12s HV [86]) | 100 | £1.00 |
| Forward Propeller (e.g. 21” [86]) | 100 | £0.50 |
| VTOL Motor (e.g. 4x Eaglepower UA90 150KV [86]) | 1,000 | £0.32 |
| VTOL ESC (e.g. 4x Hobbywing 80A 12S ESC [86]) | 100 | £3.20 |
| VTOL Propeller (e.g. 4x Eaglepower UC2480L 24” Propeller [86]) | 100 | £1.60 |
| Servo (e.g. 2x Savox SC-1256TG, 2x Savox SC-1251MG, 2x Savox SH-0263MG [86]) | 250 | £1.20 |
| Li-Po Battery (e.g. 2x Tattu HV 32000mAh 6S 10C [86]) | 500 | £1.20 |
| Base Platform (e.g. Mugin Mugin-5 Pro) | 1,000 | £5.00 |
| Autopilot (e.g. Distributed Avionics Masterless [87]) | 1,000 | £10.00 |
| Satellite Receiver (e.g. Honeywell Satcom [88]) | 1,000 | £0.50 |
| Terrestrial Mobile Network Receiver (LTE) [89] | 1,000 | £0.20 |
| Radio Comms | 1,000 | £0.20 |
| Radio Control Units (x2) | 1,000 | £0.30 |
| Ground Control System | 1,000 | £1.00 |
|  |  |  |
| **Item** | **Cost** | **Cost Basis** |
| Insurance   - Legal liability to 3^rd^ parties (bodily Injury and/or Property Damage GBP 10,000,000 each occurrence) - Noise liability (GBP 2,500 – any one occurrence and in the annual aggregate) - Invasion of privacy (GBP 2,500 – any one occurrence and in the annual aggregate)  1. Product Liability - £10 million 2. Employers’ Liability - £10 million 3. Professional Indemnity - £2 million | £2,000 | Per Year |
| Electricity [90] | £0.17 | Per kWh |
| Safety Pilot | £50 | Per Hour |
| Mission Commander | £50 | Per Hour |
| Loader/Unloader [91] | £10.26 | Per Hour |
| Delivery Driver [72] | £10.78 | Per Hour |
| Van Mileage [72] | £0.46 | Per Mile |
